# Evaluation of Daily Behavioral Patterns and Locomotor Activity in Holstein Dairy Cattle Under Different Physiological Stages and Management Factors

**DOI:** 10.3390/ani16182903

**Published:** 2026-09-15

**Authors:** Tugce Necla Selvi, Hakan Ustuner, Sena Ardicli, Emre Arslanoglu, Yaren Ozkaraca, Nursen Senturk, Abdülkadir Orman

**Affiliations:** 1 Animal Science, Faculty of Veterinary Medicine, Bursa Uludag University, Bursa 16059, Türkiye; hustuner@uludag.edu.tr (H.U.); 612454001@ogr.uludag.edu.tr (Y.O.); orman@uludag.edu.tr (A.O.); 2 Department of Genetics, Faculty of Veterinary Medicine, Bursa Uludag University, Bursa 16059, Türkiye; sardicli@uludag.edu.tr; 3 Dairysense Technology Services, Inc., Bursa 16285, Türkiye; earslanoglu@dairysense.com.tr; 4 Department of Physiology, Faculty of Veterinary Medicine, Bursa Uludag University, Bursa 16059, Türkiye; entrknursen@gmail.com

**Keywords:** behavior, welfare, Holstein, heifer, dry cows, lactation, parity

## Abstract

Precision livestock technologies play a critical role in determining animal behavior and welfare. This study aimed to determine the effects of physiological stage, parity, lactation number, and milking frequency on the locomotor, resting, and inactive time behaviors of Holstein dairy cows. The behavioral data and step counts of the animals were continuously recorded using neck collars and foot pedometers, and a total of 8698 raw daily cow-level behavioral records were analyzed. In the statistical model, the days in milk and daily milk yield were included as fixed effects. Heifers and dry cows showed greater similarity in behavioral duration than primiparous and multiparous cows. There were highly significant differences between the groups in terms of rumination, lying, standing duration, and number of steps (*p* < 0.001). In lactating cows, no statistically significant differences were found in behavioral durations according to lactation number (*p* > 0.05); however, milking frequency had a significant association across groups (*p* < 0.001). This study highlights the determining and interrelated effects of physiological stages and management factors on the locomotor and resting behaviors of cattle, thereby providing a behavioral framework for interpreting sensor-derived data in precision livestock farming and supporting data-driven herd management.

## Simple Summary

Improving the welfare of farm animals and better understanding their behavioral differences are vital for sustainable livestock farming. However, there is still limited knowledge of how dairy cows’ daily time budgets are influenced by factors such as parity, milk yield, and different management practices. This study used smart neck and leg sensors to continuously monitor the daily behavior patterns of Holstein cows. The analysis clearly separated the animals into distinct groups based on different physiological stages, revealing that heifers were far more active and exploratory, spending significantly more time standing and walking. Lactating cows naturally spent more of their day feeding and ruminating to support the high energy demands of milk production. Furthermore, increasing the daily milking frequency from twice to three times a day increased milk production and the time the cows spent feeding whilst reducing their inactive resting time duration due to increased movement and activity. This study clearly demonstrates that factors such as lactation number, parity, physiological stage, and milking frequency significantly influence the locomotor and activity patterns of dairy cows. Our study provides an essential foundation for investigating the long-term effects of behavioral changes in heifers on future milk yield and reproductive performance.

## 1. Introduction

Livestock production, particularly in the dairy sector, remains a cornerstone of global food security and economic sustainability. With the global population projected to exceed 9 billion by 2050, demand for high-quality milk and dairy products is expected to increase by around 70% [1]. Meeting this demand requires not only an increase in the number of animals but also a significant improvement in per-animal productivity through modernized management strategies. In growing cattle industries, such as in Türkiye, the transition from traditional small-scale farming to industrial-scale modernization has underscored the necessity for standardized, data-driven management models. Within these systems, animal behavior is a primary indicator of dairy cattle’s welfare, health, and physiological status. These animals follow a pattern in which essential activities, specifically lying, rumination, and feeding, are precisely balanced. High-yielding Holstein cows, for instance, typically require 10–12 h of daily lying time to optimize rumination efficiency and mammary blood circulation [2,3,4,5,6,7].

In monitoring behavioral changes in cows, while a significant amount of research in the field focuses on early disease detection, there is growing interest in elucidating the zootechnical factors that shape specific behavioral expressions [4,8]. Within this context, time budgets are best analyzed as an integrated set of behavioral parameters monitored over continuous 24 h cycles. Dairy cows demonstrate distinct diurnal patterns in feed intake that are closely synchronized with technical interventions, such as milking schedules and the delivery of fresh rations [9]. Furthermore, each individual’s behavioral patterns reflect the fundamental circadian rhythms that determine the animal’s routine [10,11]. Identifying significant alterations in these cycles provides a robust diagnostic framework for the automated detection of estrus and various subclinical health disorders. Consequently, monitoring fluctuations in daily behavioral routines is pivotal for timely identification of subclinical health issues and enhancing reproductive management efficiency [12]. Although it is traditionally assumed that rumination occurs preferentially during lying, empirical validation of this specific association remains limited. Furthermore, lying behavior in lactating dairy cattle follows a noticeable daily rhythm that operates inversely to feeding patterns [13,14]. Such interdependent behaviors require careful observation due to their complexity.

However, as dairy operations scale toward industrial intensification, providing individual attention to each animal becomes increasingly challenging. Traditional monitoring, often limited by labor constraints and the subjectivity of visual observation, is increasingly being replaced by precision livestock farming (PLF) systems. These technologies digitize the behavioral time budget, providing an objective and continuous record of an animal’s daily life. By utilizing high-resolution sensors such as accelerometers and rumination monitors, management can transition from reactive protocols to proactive, data-driven strategies. The key advantage of these digital tools is their ability to objectively and continuously quantify behavioral patterns. Such early detection is a prerequisite for optimizing metabolic efficiency and maintaining high welfare standards.

The fundamental research problem is the limitation in accurately detecting automated behavioral deviations due to the absence of well-defined, physiology-specific reference standards. Because sensor metrics are highly sensitive to routine management and metabolic demands, the lack of standard reference profiles for healthy animals creates a critical challenge in differentiating normal behavioral variability from actual welfare compromises. Despite the increasing adoption of PLF technologies, a significant knowledge gap persists in understanding how baseline behavioral patterns vary across physiological stages and productivity levels under commercial field conditions. While existing research has predominantly focused on isolated health events or specific diseases, the comparative dynamics of daily activity between heifers and cows, particularly across varying milk yield groups, remain underexplored.

This study was designed to bridge this gap by establishing the daily activity and behavioral parameters of Holstein heifers and dry and milking cows at different yield levels using advanced monitoring systems. Our objective was not only to characterize behavioral differences among physiological groups but also to establish biologically relevant reference profiles that may improve the interpretation of sensor-derived behavioral data. Specifically, it aimed to clarify time budget allocations across distinct states. From a practical standpoint, our goal is to enable herd managers, veterinarians, and researchers to refine monitoring algorithms, optimize daily management routines, and enhance automated welfare assessment.

## 2. Materials and Methods

### 2.1. Study Area and Animals

**Figure 1:**
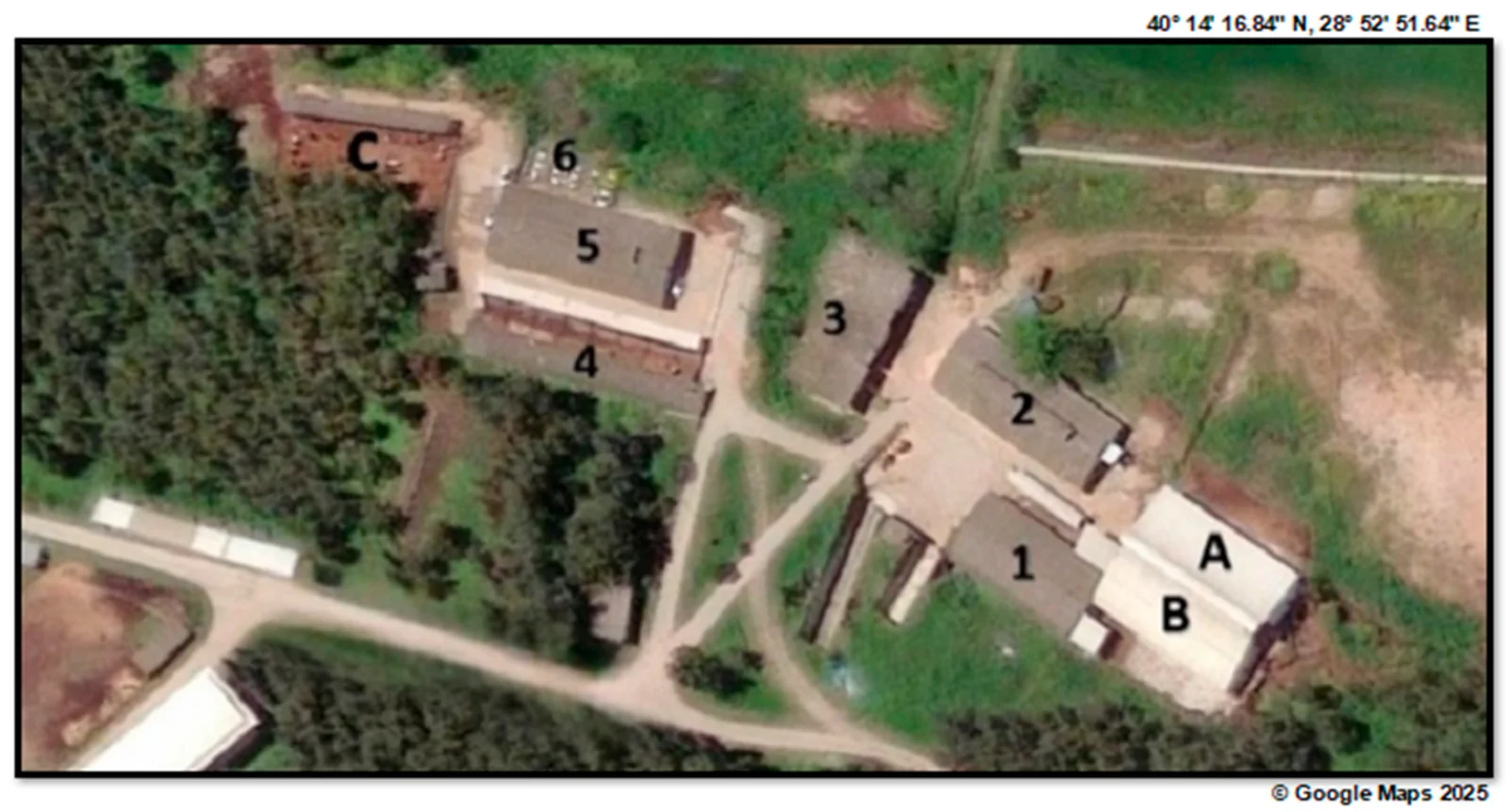
Plan of Animal Health and Animal Production Research and Application Center. (A) Free-stall barn for cows with milk yield > 25.41 L, (B) free-stall barn for cows with milk yield < 25.41 L, and (C) open barn for heifers and dry cows. 1: Milking parlor (2 × 4 double-sided herringbone), 2: cow barn building, 3: feed warehouse, 4: barn for bulls, 5: building with individual calving pens, and 6: individual calf pens.

This study was conducted between 1 September 2024, and 30 May 2025, at the Bursa Uludag University Faculty of Veterinary Medicine, Animal Health and Animal Production Research and Application Center (40°14′16.84” N, 28°52′51.64” E), Türkiye (Figure 1). The research focused on behavioral records; therefore, the sample sizes (n) and animal numbers reported reflect the initial group assignments. Throughout the study period, dynamic transitions occurred within the herd, including cows moving from the dry period to lactation, cows transitioning from lactation to the dry period, and heifers calving to the primiparous group.

### 2.2. Housing, Management, and Feeding

Lactating cows (primiparous and multiparous) were housed in a free-stall barn consisting of two pens (A and B) separated by a drive-through feed alley (2). Each pen featured a 33 m long feed line, 44 headstalls (75 cm each), and 44 cubicles equipped with polyurethane foam mattresses. During the study period, the average stocking density was 2.75 lying stalls per cow, ensuring optimal animal welfare. Walking alleys consisted of solid, grooved concrete flooring and were cleaned by automatic scrapers three times daily at 8 h intervals, with each cycle lasting 37 min. Heifers and dry cows (C) were housed in an open barn specifically designed to block prevailing northern winds while providing maximum natural ventilation. This facility utilized a deep-bedding system with soil (20–23 cm depth), regularly managed to maintain a dry resting surface. All animals were fed a total mixed ration (TMR) ad libitum twice daily and had unrestricted access to water via self-filling troughs. Regarding milking frequency, one lactating group was milked twice daily, while the other was milked three times daily (Dairysense, Bursa, Türkiye). Milking frequency was not established via randomized experimental allocation; rather, during the longitudinal trial period, the farm management transitioned the entire lactating herd from a 3×-daily to a 2×-daily milking schedule. Consequently, behavioral comparisons between milking frequencies reflect herd-level responses to this routine management shift under commercial conditions.

The TMR was offered ad libitum and delivered twice daily at 08:00 and 16:00 h. The ratio was calculated using dry matter, and its components are shown in Table 1.

### 2.3. Behavioral Data Collection

Behavioral and locomotor data were continuously recorded 24 h/day using neck-mounted collars and leg-mounted pedometers. The sensor hardware continuously captured behavioral dynamics, aggregated the measurements internally, and transmitted 24 distinct hourly data packets per device each day. Data transmission was managed via a long-range (LoRa) wireless communication protocol to prevent packet collision. The on-farm infrastructure comprised two LoRa receiver antennas (one serving lactating barns A and B, and one serving barn C for heifers and dry cows), a central communication box, and an automated parlor interface integrating 8 individual electronic milk meters and animal identification units. All sensor-derived behavioral data, activity information, and individual milking parameters were synchronized and stored in the central herd management software (Dairysense, Bursa, Türkiye) for subsequent processing and analysis.

Lying duration (min/d) was recorded by leg pedometers based on posture. Periods characterized by prolonged immobility without active feeding or rumination neck movements were captured by neck collars and operationalized as inactive resting time (min/d).

A total of 8698 raw cow-days of behavioral observations (daily cow-level records) were evaluated, categorized as follows: heifers (459 records; *n* = 7), dry cows (748 records; *n* = 12), primiparous cows (3175 records; *n* = 15), and multiparous cows (4315 records; *n* = 19). Detailed descriptive statistics of the lactating groups, including days in milk (DIM), parity, daily milk yield (DMY), lactation milk yield, previous lactation milk yield, and peak yield, are presented in Table 2.

### 2.4. Environmental Monitoring

To minimize heat stress, the free-stall barn was equipped with an automated active cooling system and fans (DeLaval, Tumba, Sweden). Microclimatic conditions were monitored using a calibrated data logger (Model V3.3, Hasvet, Istanbul, Türkiye) positioned at the animal level (~1.5 m height). The device recorded ambient temperature (T, °C) and relative humidity (RH, %) at 15 min intervals. These data were used to calculate the Temperature–Humidity Index (THI) according to the following formula [14]:

THI = (1.8 × T + 32) − [(0.55 − 0.0055 × RH) × (1.8 × T − 26)]

For environmental summarization, 15-min recordings were averaged to obtain daily values, which were used to determine the mean ± SEM and range (min–max). Microclimatic parameters recorded between September 2024 and May 2025 are summarized in Table 3.

### 2.5. Statistical Analyses

Statistical analyses were performed using linear mixed-effects models fitted by restricted maximum likelihood (REML) estimation in Minitab Statistical Software (Version 17.1.0, Minitab LLC, State College, PA, USA). Each behavioral trait, including rumination, feeding, locomotor activity, inactive resting time, daily step count, lying time, standing time, and bouts, was analyzed independently.

The statistical model was specified as follows:

*Y_ijklmn_ = μ + Ai + Bj + Ck + Dl + β*_1_* DIM + β*_2_* DMY + Animal_l_ + ε_ijklmn_*

where *Y_ijklmn_* is the observed behavioral trait, μ is the overall mean, *A_i_* is the fixed effect of housing system/floor type, *B_j_* is the fixed effect of milking frequency, *C_k_* denotes the fixed effect of lactation number, and $\beta_{1}$ and $\beta_{2}$ are the regression coefficients associated with the continuous covariates days in milk (DIM) and daily milk yield (DMY), respectively. *Animal_l_* is the random effect of the *l*th individual animal, assumed to be independent and normally distributed as $u_{l}∼N(0,\sigma_{u}^{2})$, and $\varepsilon_{ijklmn}$ is the residual error term $\left(\varepsilon_{ijklmn}∼N(0,\sigma_{e}^{2})\right)$.

Because multiple daily behavioral observations were obtained from each animal throughout the study period, individual animal identity was included as a random effect to account for the hierarchical and repeated-measures structure of the dataset. Accordingly, the individual animal, rather than each daily sensor-derived record, was considered the independent biological unit. This random-effect specification accounted for the clustering and non-independence of repeated observations within the same animal and prevented individual daily records from being treated as independent biological replicates, thereby reducing the risk of pseudo-replication. Housing system/floor type, milking frequency, and lactation number were specified as categorical fixed effects, whereas DIM and DMY were included as continuous fixed covariates (Appendix A). No additional explicit serial temporal covariance structure (e.g., first-order autoregressive [AR(1)]) was imposed between consecutive daily observations. The primary objective of the analysis was to characterize overall behavioral differences associated with physiological and management-related factors rather than to model individual temporal trajectories or day-to-day changes in behavior. Thus, repeated observations were accommodated at the animal level using a random-effects structure. Nevertheless, potential residual serial correlation between successive observations beyond that captured by the animal-level random effect cannot be entirely ruled out and should be considered when interpreting the longitudinal behavioral data.

The multivariable mixed-model framework enabled estimation of the independent effects of housing system, milking frequency, lactation number, days in milk, and daily milk yield while controlling for individual-animal variability. Continuous covariates (DIM and DMY) were included as linear covariates in the mixed-effects model. The statistical significance of fixed effects was assessed within the linear mixed-model framework, whereas variance components associated with random effects were estimated using REML. Whenever a significant fixed effect was detected, pairwise comparisons among least-squares means were performed using Tukey’s honestly significant difference (HSD) adjustment to control the family-wise Type I error rate.

Model adequacy was evaluated through graphical assessment of standardized residuals to verify the assumptions of normality, homoscedasticity, and independence of residuals. Statistical significance was declared at *p* < 0.05, whereas *p* < 0.01 and *p* < 0.001 were considered to indicate moderate and strong statistical evidence, respectively.

Principal Component Analysis (PCA) was performed to compare behavioral profiles of animal groups (heifer, dry, primiparous, and multiparous) and visualize complex relationships with OriginPro software (Versions 2024 and 2025b, OriginLab Corp., Northampton, MA, USA). The multivariate dataset was normalized and transformed into principal components (PCs). A biplot was generated to illustrate group clusters and the impact of specific parameters on these separations. Additionally, 95% confidence ellipses were used to define the boundaries of each group.

## 3. Results

The daily time budgets and activity levels of heifers and Holstein cows showed significant variations across different physiological stages and production levels. These variations reflect the animals’ behavioral adaptations to their specific physiological requirements. The comparative descriptive statistics for these behavioral parameters are detailed in Table 4.

The data revealed that, while certain behavioral parameters, such as feeding duration, locomotion activity, and inactive resting time, remained statistically unaffected by the physiological stage or production level (*p* > 0.05), rumination time showed a significant variation (*p* < 0.05). On the other hand, locomotor measurements demonstrated highly significant differences among the evaluated groups (*p* < 0.001). Regarding pedometric activity, heifers (1510.49 ± 103.20 no/d) and primiparous cows (1453.75 ± 94.53 no/d) exhibited remarkably higher daily step counts than dry cows (1005.35 ± 99.83 no/d) and multiparous cows (909.20 ± 93.27 no/d) (*p* < 0.001). This active behavior was further reflected in the standing patterns, where heifers spent the maximum time standing (798.66 ± 25.69 min/d), followed by primiparous (694.15 ± 22.01 min/d), multiparous (612.35 ± 21.48 min/d), and dry cows (543.78 ± 24.84 min/d), establishing a highly distinct separation among all four groups (*p* < 0.001). However, lying durations followed a contrasting pattern, with primiparous cows (674.04 ± 38.62 min/d) and heifers (659.96 ± 40.66 min/d) allocating significantly more time to lying than multiparous (587.26 ± 38.32 min/d) and dry cows (548.70 ± 39.73 min/d) (*p* < 0.001). Furthermore, the frequency of bouts was modulated by the production group (*p* < 0.05), with primiparous cows changing their activity most frequently (12.49 ± 0.67 no/d), which differed significantly (*p* < 0.05) from multiparous cows (10.83 ± 0.66 no/d). In contrast, heifers and dry cows displayed intermediate values.

Descriptive statistics and comparative findings regarding the daily behavioral parameters of dairy cows classified by lactation number are presented in Table 5. Statistical analysis revealed that parity or lactation number did not have a statistically significant effect on most of the behavioral parameters evaluated (*p* > 0.05). No statistically significant differences were found between cows in their first, second, third, or fourth lactation periods in terms of basic activities, including daily rumination time (482.70 ± 32.53, 500.28 ± 37.11, 541.55 ± 42.71, and 432.08 ± 62.47 min/day, respectively) and feeding time (107.66 ± 30.71, 133.09 ± 40.61, 197.29 ± 51.57, and 114.16 ± 83.88 min/day, respectively). Similarly, locomotor activity, inactive resting time, steps, lying time, and standing time remained statistically unchanged across all lactating groups (*p* > 0.05). In contrast, the daily frequency of bouts was significantly influenced by the number of lactations (*p* < 0.001). Cows in their third lactation (12.61 ± 1.68 times/day) showed statistically significantly more bouts than cows in their second lactation (7.60 ± 1.00 times/day) (*p* < 0.001). In contrast, cows with first and fourth parity (first and fourth lactations) were in intermediate ranges without forming a statistically significant separation from the other groups (11.44 ± 0.84 and 9.65 ± 3.73 times/day, respectively).

The descriptive statistics and comparative results regarding the associations between milking frequency (MF) and the daily behavioral parameters of lactating cows are detailed in Table 6. Remarkably, statistically significant differences were observed across all evaluated behavioral activities (*p* < 0.001). Cows milked three times a day (MF × 3) exhibited substantially higher digestive and active behaviors than those milked twice a day (MF × 2), as evidenced by prolonged rumination (509.28 ± 31.22 vs. 489.35 ± 31.03 min/d) and feeding durations (152.01 ± 29.60 vs. 115.92 ± 29.50 min/d). This heightened activity in the MF × 3 group was further supported by increased locomotion activity (90.14 ± 10.98 min/d) and a greater daily step count (1171.03 ± 226.31 no/d), whereas the MF × 2 cows recorded lower values of 77.27 ± 10.94 min/d and 1045.13 ± 225.76 no/d for the respective traits. Conversely, cows managed with a lower milking frequency (MF × 2) spent significantly more time resting, showing longer inactive periods (309.40 ± 30.39 vs. 265.75 ± 30.58 min/d), extended lying periods (685.24 ± 70.30 vs. 603.94 ± 70.46 min/d), and higher standing times (739.05 ± 66.91 vs. 701.82 ± 67.07 min/d). Additionally, the frequency of daily bouts, although numerically close, remained highly significant (*p* < 0.001), with the MF × 2 group changing position slightly more often (10.99 ± 0.96 no/d) than the MF × 3 group (10.88 ± 0.97 no/d).

## 4. Discussion

**Figure 2:**
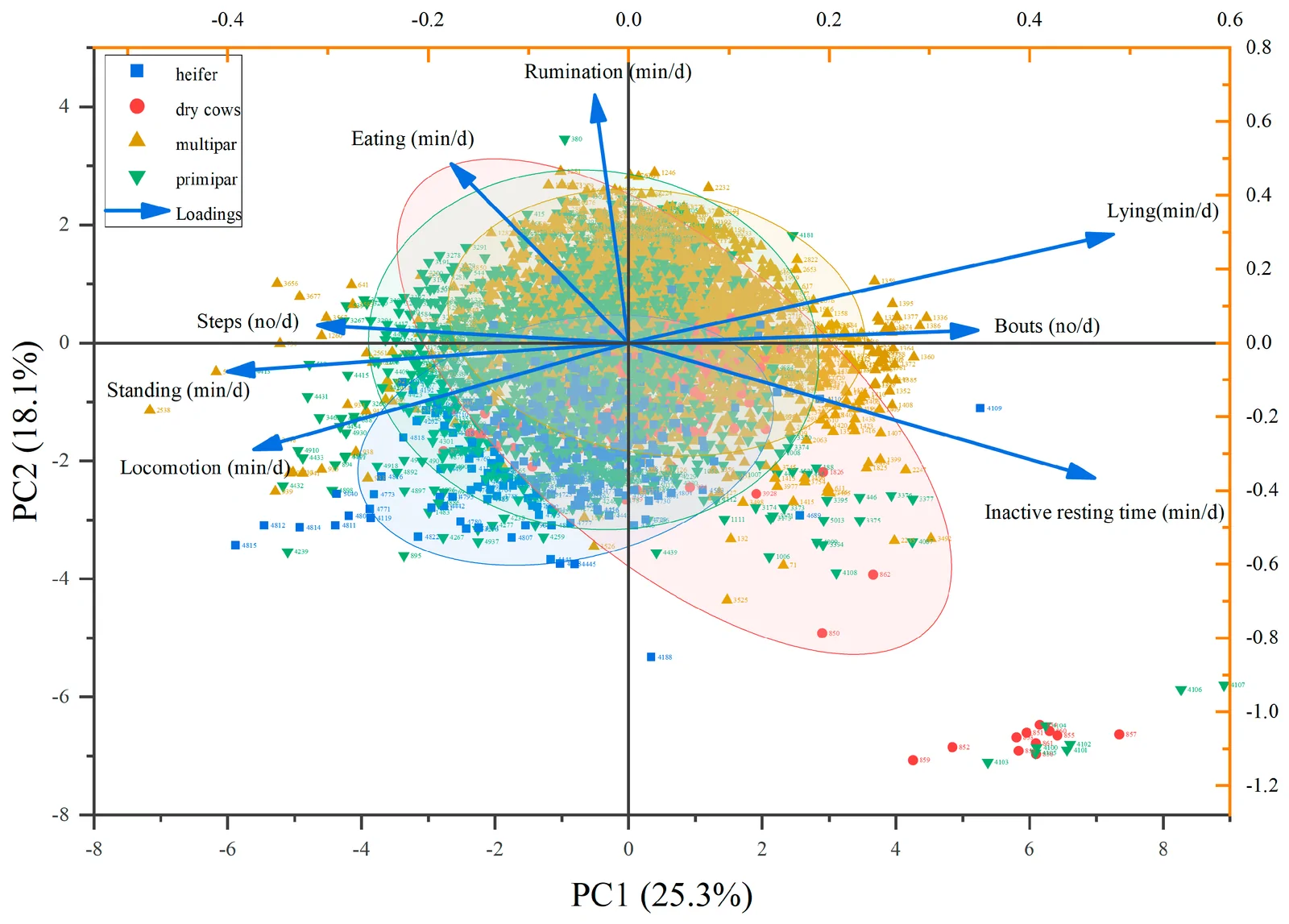
Principal Component Analysis (PCA) biplot of behavioral parameters for cows in different groups (heifers, dry cows, primiparous, and multiparous).

Precision livestock farming (PLF) technologies generate large datasets of behavioral data that require comprehensive analysis to yield meaningful biological predictions. The results of the Principal Component Analysis (PCA), which was conducted to reveal the similarities and differences in the behavioral profiles of all experimental groups, are shown in Figure 2. Concerning the PCA model, the first two principal components (PC1 and PC2) explained 43.5% of the total variance (25.3% and 18.2%, respectively), indicating that these components adequately reflected the variability and group-specific differences in the original dataset. Analysis of the score plot revealed a distinct spatial separation between heifers and dry cows; whilst this indicated behavior patterns specific to these physiological stages, primiparous and multiparous cows formed overlapping clusters, reflecting similar behavioral patterns. The intensity of vector directions further highlighted these characteristics: high activity parameters, such as standing time and step count, showed a positive correlation on the right axis and were predominantly associated with the heifer group. This is consistent with previous findings that younger and non-lactating animals generally exhibit more movement and exploratory behavior [15,16,17,18]. In contrast, activity-related parameters such as lying down, inactive resting time, and bouts clustered on the opposite axis, demonstrating a biological balance with activity levels. Lactation groups (primiparous and multiparous), consistent with the increased metabolic demands and homeostatic changes characteristic of lactation, were primarily associated with vectors related to rumination and feeding [19,20,21,22,23]. Narrow angles observed between specific behavioral vectors indicate strong positive correlations. At the same time, the differing orientations of standing and lying down durations confirm the biological antagonism between these behaviors within the dairy cow’s time budget [24,25]. It is important to note that the PCA model represents patterns in behavioral characteristics across the physiological stages of heifers and cows. Hence, we did not include the random animal factor in the PCA model.

Beyond visualizing group separation, the PCA findings indicate that behavioral traits should not be interpreted as isolated variables but rather as components of an integrated behavioral phenotype. The clustering of animals according to physiological stage suggests that multiple behavioral parameters respond coordinately to changes in metabolic demand and production status, highlighting the biological complexity underlying sensor-derived behavioral data.

Rumination is a unique function specific to ruminants, defined as regurgitating fibrous nutrients from the stomach back into the mouth, chewing them again, moistening them with saliva, and ingesting them; it facilitates the passage of roughage through the digestive system. This activity not only facilitates digestion by reducing feed particle size but also maintains stomach pH (5.5–6.5), an ideal level for microbial activity, through the secretion of saliva [26,27,28,29]. Cattle ruminate for 25–80 min per kilogram of roughage consumed, and healthy, mature dairy cows ruminate for 7–8 h a day. It has been reported that the average rumination and feeding time in dairy cows is 463 min per day for primiparous cows and 522 min per day for multiparous cows, which is consistent with our study results [28,30,31]. A higher rumination time can be explained by the animal’s increased metabolic requirements and larger digestive system. Cows ruminate in 10 to 17 cycles throughout the day; they chew 30–60 times per cycle and are usually positioned lying on their left side. However, while environmental conditions such as heat stress increase the frequency of standing rumination, adverse factors such as discomfort, pain, and hunger experienced during milking, as well as different housing conditions, directly affect this behavior [32,33,34,35]. Locomotor activity and step count were high in heifers, and the greater activity of younger animals contributed to these behavioral values being more favorable. In our study, the effect of lactation number on locomotor activity was also clearly observed. Accordingly, Brzozowska et al. [15] reported the daily step counts of lactating Holstein cows during their first, second, and third lactations, as well as following lactations, as 1525, 1495, and 1541, respectively. Although the values obtained for the first and third lactations in that study are higher than our values, they show a similar pattern; in contrast, the activity levels of cows during the second lactation are lower than those found in our study. It is suggested that these differences stem from variations in herd management strategies, housing conditions, and the animals’ physiological adaptation during the postpartum period. Inactive resting time duration was determined to be highest in dry cows. As in other mammalian species, including humans, an extended period of inactivity in pregnant animals is considered normal due to physiological requirements and metabolic changes associated with gestation [36]. In another study, primiparous cows exhibited longer daily durations and more frequent lying-down behavior than multiparous cows (for sleep: 70.0 min/day, 11.5 times/day and 49.4 min/day, 8.3 times/day, respectively; for lying down: 848. 2 min/day, 13.6 times/day and 786.8 min/day, 11.3 times/day), stemming from physiological needs such as the continuation of the growth period and the associated release of anabolic hormones rather from than discomfort or stress [37,38,39]. In line with this, the inactive resting time and lying-down durations obtained in our study are consistent with these values reported in the literature. Studies on this behavior are quite limited; therefore, the inactive time values in our results are expected to provide a crucial reference baseline for future research.

Milking frequency was associated with highly statistically significant variations (*p* < 0.001) in all behavioral parameters assessed between the groups milked twice and three times. As is well documented in the literature [40,41], an increase in milking frequency leads to a marked increase in milk yield; consequently, the finding that feeding and rumination times were higher in the group milked three times is fully consistent with physiological expectations. On the other hand, a reduction in inactive time and lying-down duration in parallel with milking frequency, coupled with an increase in daily step count, has been identified as an interrelated dynamic directly linked to milking parlor traffic and increased metabolic activity. These findings further emphasize that behavioral alterations detected by precision livestock monitoring systems should always be interpreted within the context of herd management practices. Otherwise, normal behavioral adaptations associated with management interventions may be incorrectly classified as indicators of disease or impaired welfare.

Our study has some limitations. Firstly, the heifer (*n* = 7) and dry cow (*n* = 12) groups had relatively small sizes compared to the lactating groups. Due to the high locomotor activity and rapid physical transitions of young heifers, strict data-filtering protocols were implemented, and only individuals with continuous and high-precision sensor transmissions throughout the trial period were retained. Although this control ensured high-density longitudinal data across continuous 24 h cycles for each monitored animal, the behavioral profiles of heifers and dry cows should be interpreted as robust preliminary baseline observations rather than definitive population-wide standards. Future large-scale longitudinal studies involving larger groups across multiple farms are recommended to further validate these subgroup-specific behavioral dynamics.

Additionally, precise daily durations spent in the milking parlor and holding areas could not be extracted retrospectively as an independent behavioral category from the sensor database; evaluating the specific time budget trade-offs between parlor transit and other daily activities remains a focus of our ongoing behavioral trials.

This study was conducted at a single commercial farm under specific housing conditions, which may limit the generalizability of the findings on locomotor and resting behavior to different herd management and housing systems. Also, the potential seasonal effects of changes in the THI on heat stress-related behavioral patterns were not controlled for in this study. Finally, the algorithmic limitations in fully distinguishing between deep sleep and periods of immobile rest using the datasets provided by the precision livestock technologies used (sensors) should be considered. Conducting future studies with larger sample groups across different seasons and housing types will contribute to a clearer assessment of these behavioral dynamics. In addition, although individual animal variability was accounted for by including animal identity as a random effect in the mixed-effects model, future studies that incorporate explicit temporal covariance structures may further improve the characterization of longitudinal behavioral dynamics. Although individual animal variability and the non-independence of repeated observations within animals were accounted for by including animal identity as a random effect in the mixed-effects model, no explicit serial temporal covariance structure was imposed between consecutive daily observations. Therefore, residual temporal correlation beyond that captured by the animal-level random effect cannot be entirely ruled out. Because the primary objective of the present study was to characterize overall behavioral differences associated with physiological and management-related factors rather than individual day-to-day behavioral trajectories, temporal dynamics were not modeled as a separate outcome. Future longitudinal studies specifically designed to investigate temporal behavioral trajectories may benefit from incorporating explicit covariance structures, such as autoregressive models, to further characterize serial dependence between repeated observations. Furthermore, investigating the potential long-term effects of these changes in locomotor activity and behavior observed in heifers on milk yield and productivity during later stages of lactation constitutes the basis of our future research.

## 5. Conclusions

In conclusion, this study clearly demonstrates that factors such as lactation number, parity, physiological stage, and milking frequency significantly influence the locomotor and activity patterns of dairy cows. It was observed that heifers and dry cows exhibit more similar behavioral durations than primiparous and multiparous cows. These findings are further supported by bouts that reflect behavioral variation. It is thought that fundamental metabolic and physiological differences, such as lactation biology and animal age, underlie this phenomenon. Furthermore, the marked decrease in standing time from heifers to multiparous animals also supports this hypothesis. Consequently, the significant effects of animal age and lactation yield on behavioral changes and their durations are among the key findings of this study.

Furthermore, the longest inactive times observed in dry cows confirm the gestational requirements specific to mammalian biology. Among the management factors, increasing milking frequency significantly affected all behavioral parameters at a high level of statistical significance: whilst increasing feeding and rumination times in parallel with the rise in milk yield, it led to a reduction in inactive resting time, lying times, and an increase in step count due to milking traffic and metabolic activity. Given the very limited number of studies in the literature on cattle sleep and resting behavior, the data obtained in this study will not only serve as an important reference database for future research in precision livestock farming technologies but also provide a foundation for optimizing animal welfare and herd management strategies.

Consequently, these findings establish reference behavioral profiles for dairy cattle across different physiological stages and management conditions. Such baseline information may facilitate more accurate interpretation of sensor-derived behavioral data, improve precision livestock farming applications, and contribute to earlier identification of deviations associated with health, welfare, and production performance. Ultimately, this study provides direct practical benefits for herd managers and veterinarians to optimize management routines and health diagnostics while offering researchers robust reference baselines for developing more precise welfare assessment models.

## Figures and Tables

**Table 1:** Nutrient contents of the total mixed ration (TMR).

Corn Silage	61.4%
Commercial Dairy Concentrate	13.2%
Straw	10.5%
Corn Flakes	10.5%
Soybean Meal	4.4%
The nutrient composition of the TMR (on a dry matter (DM) basis)	
Dry Matter	53%
on a DM basis	
Crude Protein	16%
Neutral Detergent Fiber	29%
Non-Fiber Carbohydrates	32%
Crude Fat	6%
Calcium	0.75%
Phosphorus	0.35%

**Table 2:** Descriptive statistics for primiparous (*n* = 15) and multiparous cows (*n* = 19) included in the study.

Variable	Mean	SD	Minimum	Maximum
DIM ^1^	265.35	134.75	42.00	462.00
Lactation number	1.78	0.89	1.00	4.00
Milking frequency (no./d)	2.40	0.49	2.00	3.00
DMY (kg/d)	25.41	9.92	1.00	56.30
Lactation milk yield (kg)	6125.92	43.36	235.00	13,141.00
Previous lactation milk yield (kg/d)	9368.33	22.48	6965.00	12,240.00
Peak yield (kg/d)	39.47	9.39	16.82	49.91

^1^ Data from cows at the beginning of the study period.

**Table 3:** Microclimatic parameters (temperature, relative humidity, and THI) recorded inside the barn (September 2024, May 2025).

Parameter	Value (Mean ± SEM)	Range (Min–Max)
Temperature (°C) *	12.11 ± 0.42	1.96–27.36
Relative Humidity (RH, %) *	69.37 ± 0.92	36.27–89.99
THI	54.43 ± 0.63	38.18–75.01

* Values are calculated based on on-site data logger records.

**Table 4:** Daily activity and behavioral parameters of heifers and Holstein cows at different production levels ($\bar{x}$ ± S$\bar{x}$)

Group	Rumination (min/d)	Feeding (min/d)	Locomotion Activity (min/d)	Inactive Resting Time (min/d)	Steps (no/d)	Lying (min/d)	Bouts (no/d)	Standing (min/d)
Heifers	530.04 ± 94.74	91.18 ± 70.76	91.28 ± 26.02	273.14 ± 94.49	1510.49 ± 103.20 ^a^	659.96 ± 40.66 ^a^	11.97 ± 0.76 ^AB^	798.66 ± 25.69 ^a^
Dry Cows	441.38 ± 67.15	98.59 ± 50.71	84.40 ± 18.68	361.10 ± 66.92	1005.35 ± 99.83 ^b^	548.70 ± 39.73 ^b^	11.30 ± 0.74 ^AB^	543.78 ± 24.84 ^d^
Primiparous	502.37 ± 16.29	134.79 ± 21.18	81.26 ± 8.21	242.18 ± 14.91	1453.75 ± 94.53 ^a^	674.04 ± 38.62 ^a^	12.49 ± 0.67 ^A^	694.15 ± 22.01 ^b^
Multiparous	553.45 ± 13.06	139.87 ± 17.35	77.65 ± 6.74	257.69 ± 11.90	909.20 ± 93.27 ^b^	587.26 ± 38.32 ^b^	10.83 ± 0.66 ^B^	612.35 ± 21.48 ^c^
Significance	**<0.05**	NS	NS	NS	**<0.001**	**<0.001**	**<0.05**	**<0.001**

^a,b,c,d^ Means in the same column followed by different letters are significantly different (*p* < 0.001). ^A,B^ Means in the same column followed by different letters are significantly different (*p* < 0.05), NS: non-significant. Values in bold indicate statistical significance at *p* < 0.05.

**Table 5:** Descriptive statistics and comparative results of daily behavioral parameters in lactating cows categorized by lactation number ($\bar{x}$ ± S$\bar{x}$).

Group (Lactation Number)	Rumination (min/d)	Feeding (min/d)	Locomotion Activity (min/d)	Inactive Resting Time (min/d)	Steps (no/d)	Lying (min/d)	Bouts (no/d)	Standing (min/d)
1	482.70 ± 32.53	107.66 ± 30.71	86.35 ± 11.36	281.51 ± 31.81	1056.60 ± 253.94	559.40 ± 81.49	11.44 ± 0.84 ^b^	705.76 ± 74.82
2	500.28 ± 37.11	133.09 ± 40.61	72.24 ± 15.10	305.06 ± 35.99	988.56 ± 255.32	629.94 ± 82.43	7.60 ± 1.00 ^c^	732.06 ± 76.06
3	541.55 ± 42.71	197.29 ± 51.57	100.16 ± 19.26	236.35 ± 41.14	1134.29 ± 304.02	746.55 ± 102.07	12.61 ± 1.68 ^abc^	664.86 ± 91.48
4	432.08 ± 62.47	114.16 ± 83.88	73.44 ± 31.39	328.94 ± 59.53	947.54 ± 432.74	647.18 ± 151.79	9.65 ± 3.73 ^abc^	751.39 ± 131.66
Significance	NS	NS	NS	NS	NS	NS	**<0.001**	NS

^a,b,c^ Means in the same column followed by different letters are significantly different (*p* < 0.001). NS: non-significant. Values in bold indicate statistical significance at *p* < 0.05.

**Table 6:** Descriptive statistics and comparative results of daily behavioral parameters in lactating cows categorized by milking frequency (MF) ($\bar{x}$ ± S$\bar{x}$).

Group	Rumination (min/d)	Feeding (min/d)	Locomotion Activity (min/d)	Inactive Resting Time (min/d)	Steps (no/d)	Lying (min/d)	Bouts (no/d)	Standing (min/d)
MF × 2	489.35 ± 31.03	115.92 ± 29.50	77.27 ± 10.94	309.40 ± 30.39	1045.13 ± 225.76	685.24 ± 70.30	10.99 ± 0.96	739.05 ± 66.91
MF × 3	509.28 ± 31.22	152.01 ± 29.60	90.14 ± 10.98	265.75 ± 30.58	1171.03 ± 226.31	603.94 ± 70.46	10.88 ± 0.97	701.82 ± 67.07
Significance	**<0.001**	**<0.001**	**<0.001**	**<0.001**	**<0.001**	**<0.001**	**<0.001**	**<0.001**

Values in bold indicate statistical significance at *p* < 0.05.

## Data Availability

The datasets generated and/or analyzed during the current study are available from the corresponding author on reasonable request.

## Abbreviations

The following abbreviations are used in this manuscript:

DIM	Days in Milk
DMY	Daily Milk Yield
THI	Temperature–Humidity Index
PCA	Principal Component Analysis
MF	Milking Frequency

## Appendix A

In the statistical model developed, days in milk (DIMs) and daily milk yield (DMY) were considered as fixed effects. The results of the statistical analysis and the significance values (*p*-values) relating to the effects of these variables on all the behavioral patterns examined are presented in Table A1.

**Table A1:** Model 1: fixed effects and significance by *p*-value.

Group	Rumination (min/d)	Feeding (min/d)	Locomotion Activity (min/d)	Inactive Resting Time (min/d)	Steps (no/d)	Lying (min/d)	Bouts (no/d)	Standing (min/d)
DIM	NS	***	***	***	**	***	NS	***
DMY	***	NS	**	**	NS	NS	NS	NS

** *p* < 0.01; *** *p* < 0.001. NS: non-significant.
